# Assessment of the impact of COVID-19 on tuberculosis care at a tertiary hospital: integrating lessons from COVID-19 learned

**DOI:** 10.3389/fpubh.2025.1505914

**Published:** 2025-03-21

**Authors:** Norma A. Téllez-Navarrete, Jesús Romero-Tendilla, Alejandra Morales, Eduardo Becerril, Néstor Alvarado-Peña, Miguel A. Salazar-Lezama, Pamela Garciadiego-Fossas, Eliane Cadena-Torres, Leslie Chavez-Galan, Lucero A. Ramón-Luing

**Affiliations:** ^1^Department of Healthcare Coordination, Instituto Nacional de Enfermedades Respiratorias “Ismael Cosío Villegas”, Mexico City, Mexico; ^2^Laboratory of Integrative Immunology, Instituto Nacional de Enfermedades Respiratorias “Ismael Cosío Villegas”, Mexico City, Mexico; ^3^Microbiology Laboratory, Instituto Nacional de Enfermedades Respiratorias “Ismael Cosío Villegas”, Mexico City, Mexico; ^4^Tuberculosis Clinic, Instituto Nacional de Enfermedades Respiratorias “Ismael Cosío Villegas”, Mexico City, Mexico; ^5^Department of Surveillance Epidemiology, Instituto Nacional de Enfermedades Respiratorias “Ismael Cosío Villegas”, Mexico City, Mexico

**Keywords:** tuberculosis, COVID-19 pandemic, healthcare, epidemiology, diagnosis TB

## Abstract

**Introduction:**

During the COVID-19 pandemic outbreak in 2020 until 2023, healthcare resources dedicated to critical diseases, including respiratory conditions like Tuberculosis (TB), were significantly impacted worldwide. The Instituto Nacional de Enfermedades Respiratorias “Ismael Cosío Villegas” (INER), a leading tertiary-level hospital in Mexico City and a national reference center for respiratory diseases, was designated exclusively for COVID-19 patients during these years.

**Methods:**

This report aims to assess the pandemic’s impact on TB care at INER and propose strategies for improving TB management by integrating lessons learned from the pandemic. TB presumptive cases were reviewed between 2016-2023, covering pre-pandemic, pandemic, and post-period; the number of diagnosis tests performed and number of attending TB patients in the emergency areas, hospitalization, or outpatient consultation were analyzed. The mortality rate of patients during hospitalization was also examined.

**Results:**

Our analysis revealed that during the pre-pandemic period (2016-2019), around 1,000 TB patient consultations were managed annually across outpatient and inpatient settings, and it drastically declined in 2020, a trend that persisted through 2021 and 2022. Survival of TB patients was affected, and disruption in TB care resulted in a decrease in TB diagnoses during the pandemic and increased mortality rates among hospitalized patients during the post-pandemic period. In response to the challenges posed by the pandemic, INER adopted innovative strategies such as telehealth services and reinforced human resources dedicated to respiratory pathologies. These efforts and enhanced diagnostic testing have strengthened the hospital’s capacity to care for TB patients. The lessons learned during the pandemic have been pivotal in reshaping and improving the healthcare system’s approach to managing TB in a tertiary care setting.

## 1 Introduction

Tuberculosis (TB) is an infectious disease caused by the primary causative agent, *Mycobacterium tuberculosis* (Mtb), a member of the *Mycobacterium tuberculosis* complex, primarily affecting the lungs. TB was the most prevalent infectious disease globally in 2023 (1), and it was the leading cause of death by a single pathogen, only replaced by coronavirus disease (COVID-19) during the recent pandemic (2). Nowadays, the World Health Organization (WHO) reports that TB probably returned to being the world’s leading cause of death from a single infectious agent (1).

This infectious disease continues to be a public health problem with high morbidity and mortality in humans; in patients without anti-TB treatment, 50–60% died; 20–25% were cured spontaneously, while 10–25% continued with symptoms of TB (3). Currently, the standard anti-TB susceptible treatment lasts 6 months; however, the WHO has approved four-month regiments using rifapentine/isoniazid/pyrazinamide/moxifloxacin (4). Regarding the treatment for drug-resistant TB, the duration for patients with more extended regimens is 15–17 months after culture conversion. However, shorter regimens such as BPaL and BPaLM (6 months) have also been approved in the last years (5).

TB can be divided into TB disease (TBD) and TB infection (TBI); TBD patients exhibit symptoms; conversely, TBI patients are in a state of persistent immune response to stimulation by Mtb antigens with no evidence of clinical manifestations of TB disease. Among the major risk factors for the development of TB are HIV infection, malnutrition, excessive alcohol use, smoking, and type 2 diabetes mellitus (6, 7).

COVID-19, a disease caused by the novel coronavirus severe acute respiratory syndrome coronavirus 2 (SARS-CoV-2), was declared a pandemic by the WHO on March 11, 2020, and its end in March 2023. Nowadays, it has resulted in over 776 million confirmed cases and more than 7.1 million deaths worldwide, impacting medical services worldwide (8).

During the COVID-19 pandemic, TB, cardio-respiratory diseases, and diabetes were among the main comorbidities that influenced the severity of COVID-19 (9). Numerous cases of TB/COVID-19 coinfection were reported during this period (10–13), underscoring the importance of social factors associated with the development of both diseases, such as poverty, socioeconomic status, housing and environmental conditions, and access to healthcare. Consequently, between 2020 and 2022, it became crucial to establish measures to improve healthcare and combat malnutrition to strengthen immune systems and enhance efforts to fight TB and COVID-19 (14, 15).

Additionally, national and local health policies aimed to address various aspects of the public health emergency, focusing on preventing the spread of the virus through physical distancing and face masks. Hospital reconversion efforts were also implemented to manage COVID-19 patients and prevent the collapse of healthcare systems. Some governments assigned hospital centers to “exclusive COVID-19 patients” (16). However, this effort to mitigate the pandemic affected other patients’ healthcare, such as those with chronic diseases who requested a follow-up. WHO revealed in 2020 that almost half of the essential health services were disrupted globally, and up to the first 3 months of 2021, these disruptions had continued in 90% of countries (17).

The COVID-19 pandemic delayed care and diagnosis for other diseases, such as cancer and heart disease (18–23). For instance, the human immunodeficiency virus/acquired immune deficiency syndrome (HIV/AIDS) and TB, diseases that cause-related deaths among people with either or the two coinfections, were negatively impacted by the COVID-19 mainly on TB case detection and HIV testing (24–29).

The Global Tuberculosis Report 2022 indicated that the COVID-19 pandemic seriously affected access to TB diagnosis and treatment, revealing a setback in the progress of TB control worldwide (5); however, the last WHO global report of 2023 (30) informed a major global recovery in the number of people diagnosed with TB and treated in 2022 after 2 years of COVID-related disruptions. Although efforts have begun to reverse or moderate the damaging impact of the pandemic on the number of people dying from or falling ill with TB, the effects are still significant.

The 2023 report indicated that the number of people newly diagnosed with TB was 7.5 million in 2022, representing the highest figure since monitoring began in 1995, above the pre-COVID baseline (and previous historical peak) of 7.1 million in 2019, and up from 5.8 million in 2020 and 6.4 million in 2021 (5, 30). The number in 2022 probably includes a sizeable backlog of people who developed TB in previous years but whose diagnosis and treatment were delayed by COVID-related disruptions that affected access to and provision of health services. Fortunately, the number of death cases decreased, perhaps because health centers resumed care for TB patients, including monitoring and adherence to the patient’s treatment.

The mortality and incidence increased; TB incidence has increased from the lowest level in 2019 with 7,100,000 vs. 10,600,000 in 2021 and 2022, and 10,800,000 in 2023, whereas mortality showed the lowest levels in 2018 and 2019 with 1,200,000 vs. 1,600,000 in 2021 and decreasing in 2022 and 2023 (Table 1).

**Table 1:** Global Incidence and Mortality of TB from 2016 to 2023.

Year	Incidence of TB^1^ (number cases)	Mortality of TB^1^ (number cases)
2023	10,800,000	1,250,000 (161,000 HIV+)
2022	10,600,000	1,300,000 (167,000 HIV+)
2021	10,600,000	1,600,000 (187,000 HIV+)
2020	9,900,000	1,500,000 (214,000 HIV+)
2019	7,100,000	1,200,000 (208,000 HIV+)
2018	10,000,000	1,200,000 (251,000 HIV+)
2017	10,000,000	1,300,000 (300,000 HIV+)
2016	10,400,000	1,300,000 (207,000 HIV+)

^1^Number of yearly cases reported by WHO (1). Numbers in parentheses are indicated for people living with HIV (HIV-positive).

Therefore, it is currently urgent to focus on establishing new health policies to reverse the downward trend in TB diagnoses and achieve the goals of the EndTB program. In this study, we analyzed the prevalence and surveillance of healthcare services provided to TB patients treated at the Instituto Nacional de Enfermedades Respiratorias (INER), a tertiary care facility in Mexico City and the leading national reference center for respiratory diseases. During the pandemic, the Mexican Health Ministry restricted INER’s services to prioritize COVID-19 care. Finally, using the analyzed pre-and post-pandemic data, we discussed established strategies at INER to enhance the management and treatment of TB patients, integrating the lessons learned from the pandemic.

## 2 Materials and methods

### 2.1 Study setting

This observational and retrospective study, conducted at the National Institute of Respiratory Diseases Ismael Cosío Villegas (INER) in Mexico City, Mexico, aims to describe the impact of the COVID-19 pandemic on the healthcare of TB patients.

The INER is a tertiary care center in Mexico City, built-in 1936, to attend to TB patients. Currently, it is a national reference for the care of patients with pathologies involving the respiratory system. However, on February 28, 2020, the Minister of Health reported the first case of SARS-CoV-2 in Mexico that was diagnosed and hospitalized at the INER. Eventually, the institution was converted to an exclusive center for attending COVID-19 patients from March 2020 to the end of 2021, disrupting attention for other diseases, including reduced attendance for TB patients. Some TB patients were only attended by outpatient or video call; patients with no COVID-19 were evaluated and posteriorly transferred to other general hospitals. The patients were isolated in the severe cases where they could not be moved.

### 2.2 Study populations and data collection

The Statistics and Epidemiological Surveillance Departments provided administrative data from January 2016 to December 2023. From the database provided, patients with presumptive TB or TB diseases were included and registered during their medical assessment during their stay in the emergency area, hospitalization, or outpatient consultation. The data corresponding to diagnostic test studies for TB requested in the pre-pandemic and post-pandemic from 2019 to 2023 in the INER Microbiology Laboratory was also presented.

### 2.3 Definitions relevant to our study are described

For this study, subjects were categorized according to the health care attention in the following areas of our center.

Outpatient Department: this is where consultations are given, and patients receive diagnosis and treatment but do not stay overnight. Depending on the manifested diseases, patients can mainly be assisted two or more times per year. For patients with sensitive TB, follow-up is recommended at 2, 4, and 6 months after diagnosis. MDR-TB patients have an appointment each month for evaluation. Patients in this area can attend specialty consultations such as pneumology, infectious diseases specialist, internal medicine, nutrition, nephrology, or psychology.

Emergency Room (ER): it is where patients with a condition that endangers their lives are immediately treated. This area receives severe TB cases that are referred from other health units.

Hospitalization: this is the area where the patients are sent or admitted to the hospital; in fact, the patient requires admission as an inpatient and usually requires an overnight stay. The length of stay varies according to the patient’s pathology. In the case of TB patients, patients with a severe presentation of TB, MDR-TB, or adverse reactions to anti-TB drugs are hospitalized and require surveillance for adverse events.

TB patient: a person who is receiving care for TB diseases.

Presumptive TB: a person who presents symptoms or signs suggestive of TB.

Pulmonary tuberculosis (PTB): any bacteriologically confirmed or clinically diagnosed case of TB involving lung parenchyma or the tracheobronchial tree, including tuberculous intrathoracic lymphadenopathy (mediastinal or hilar without radiographic abnormalities in the lungs). It is essential to mention that a person with both pulmonary TB and extrapulmonary TB is classified as having pulmonary TB.

Extrapulmonary TB: any bacteriologically confirmed or clinically diagnosed case of TB involving organs other than lungs (E.g., pleural, peripheral lymph nodes, abdomen, genitourinary tract, skin, joints, bones, meninges).

Mycobacteria culture: growing the mycobacteria on a solid or liquid medium enriched with nutrients.

GeneXpert® MTB/RR Ultra: is a rapid test nucleic acid amplification method designed as an integrated DNA extraction and real-time PCR (RT-qPCR) system that can identify *M. tuberculosis* and resistance to rifampicin.

AFB (acid-fast-bacilli): Smear microscopy is a Ziehl-Neelsen staining method, a standard laboratory method of staining smears for TB diagnosis.

### 2.4 Descriptive analysis

Data were grouped according to the diagnosis of patients with pulmonary TB and extrapulmonary diseases (PTB), pooled severe cases of TB (miliary and meningeal TB), and all test diagnostic of TB were realized in the Laboratory of Microbiology. Descriptive epidemiologic analysis and graphics were performed using GraphPad Prism Version 9.5.0 software.

## 3 Results

We analyzed the frequency of people who attended with a pulmonary and extrapulmonary TB diagnosis between 2016 and 2023. We observed that the number of attended people outpatients, ER, and hospitalizations decreased in 2020, the year of the COVID-19 pandemic, mainly during attending outpatients. Noteworthy, in 2020, our hospital was exclusive for attendance patients diagnosed with COVID-19.

### 3.1 The COVID-19 pandemic affected mainly attending outpatients

**Figure 1:**
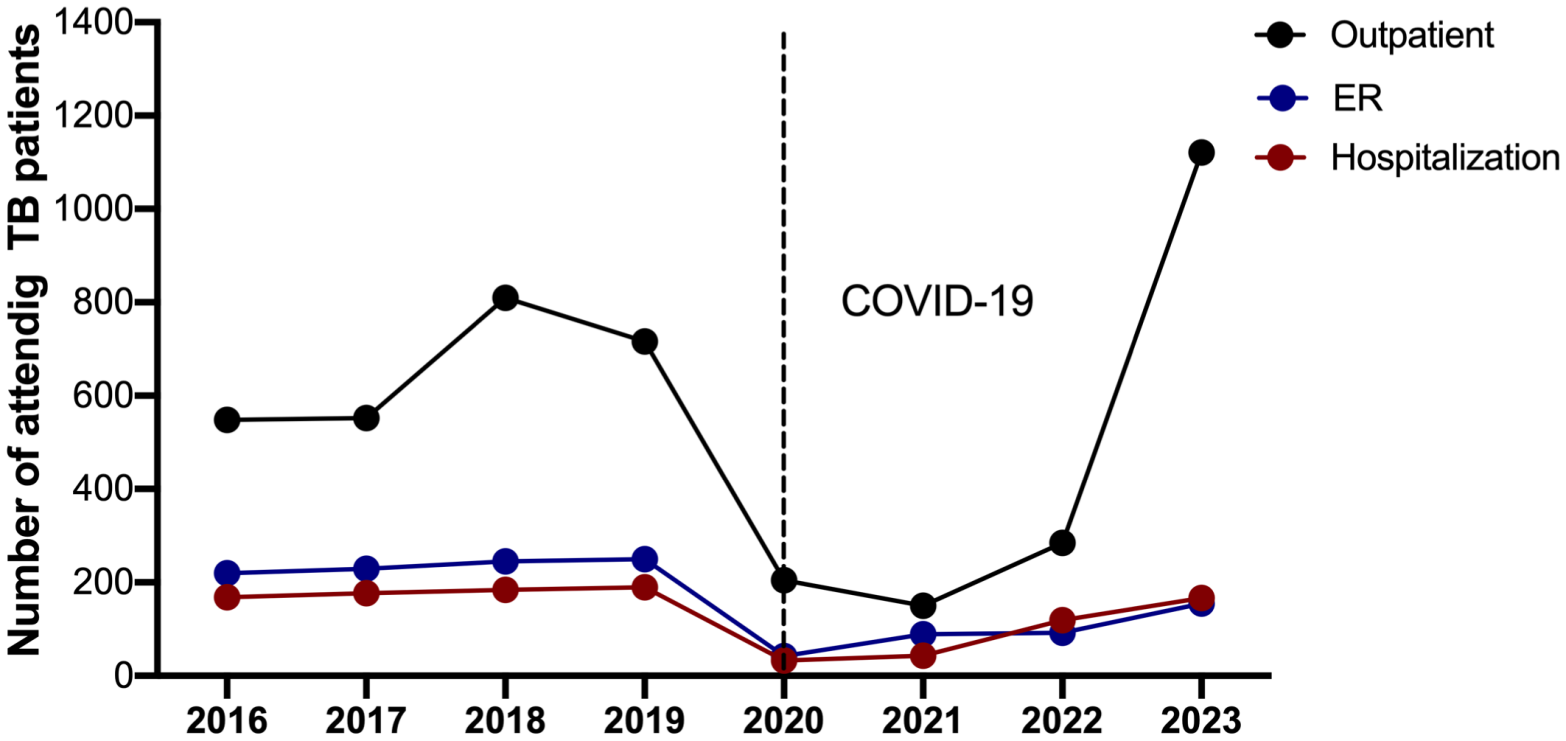
Number of attended visits of patients with pulmonary and extrapulmonary tuberculosis cases abruptly decreased in 2020 and 2021. The number of TB patients attended from 2016 to 2023 in each area is shown: outpatient, emergency room (ER), and hospitalization. The black dotted line marks the sharp decline in 2020, coinciding with the start of the COVID-19 pandemic.

In the four pre-pandemic years (2016–2019), INER attended an average per year of 656 TB patients as outpatients, 236 patients in the ER, and 179 patients hospitalized (Figure 1; Table 2). In total, approximately 1,000 TB cases were treated annually. It is important to note that this number reflects the total number of medical consultations, not individual patients; this means that one patient may have been counted multiple times if they were transitioned from one area, such as from outpatient care to hospitalization, based on the criteria set by medical specialists.

**Table 2:** The number of registers of attending TB patients between 2016 and 2023.

Area	2016	2017	2018	2019	2020	2021	2022	2023
Outpatient	548	552	810	716	205	150	285	1,121
ER	220	229	245	250	42	89	92	155
Hospitalization	168	177	184	190	33	43	119	167
TOTAL^1^	936	958	1,239	1,156	280	282	496	1,443

^1Number of registered TB patients per year in each area of attention at the hospital, including pulmonary and extrapulmonary tuberculosis cases.^

Figure 1 shows that in 2020–2021, there was a dramatic disruption in the attention given to TB patients. As is indicated in Table 2, the total number of TB patients who attended was similar (280 and 282, respectively). In 2020, 205 outpatients, 42 in the ER, and 33 hospitalized were attended; it was similar in 2021; there was an average per year of 150 outpatients, 89 in the ER, and 43 hospitalized.

However, after the hospital conversion in 2022, the last pandemic year, patient care in the outpatient area increased.

### 3.2 Increasing deaths in hospitalized TB patients during the post-COVID-19 pandemic

**Figure 2:**
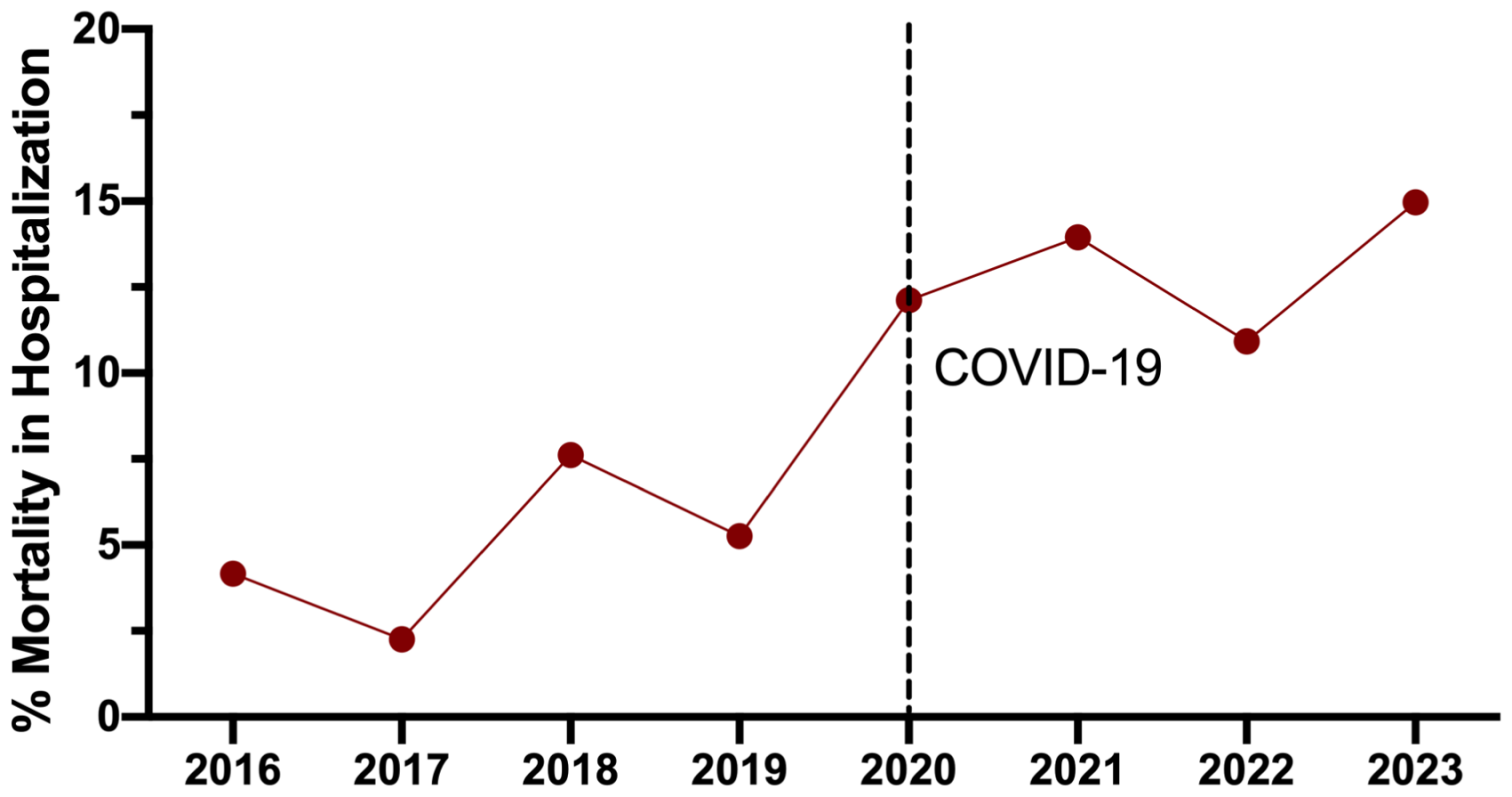
Annual mortality percentage of hospitalized TB patients, comparing pre-and post-COVID-19 pandemic periods. The black dotted line delimits the start of the COVID-19 pandemic in 2020 year.

To assess mortality among hospitalized patients, the number of deaths in the hospitalization area (Table 3) was compared to the number of hospitalized TB cases (Table 2), and the death percentage is presented in Figure 2. During the pre-pandemic years (2016–2019), the average percentage of deaths in the hospitalization area maintained an average of 4.4%. However, this rate tripled in 2020, reaching 12.1%, reflecting the emergency’s impact on managing hospitalized TB patients. This percentage has been growing; it was 13.9% in 2021 and 10.9% in 2022, but in 2023 it increased to 14.9%. Finally, the TB-reported deaths during the pandemic and post-pandemic during 2020–2023 were 15 women (31.25%) and 33 men (68.75%).

**Table 3:** Number and percentage of annual mortality of TB during hospitalization.

Area	2016	2017	2018	2019	2020	2021	2022	2023
Number of patients with TB^1^	168	177	184	190	33	43	119	167
Number of Deaths TB	7	4	14	7	4	6	13	25
Percentage of Mortality (%)	4.1	2.2	7.6	3.7	12.1	13.9	10.9	14.9

^1^Number of cases reported per year in hospitalization area. Data are shown as the number of patients or deaths and percentage (%).

Regarding TB/COVID-19 co-infection, a total of eight cases were recorded in the ER and hospitalization; among them, only two patients were diagnosed during their hospital stay. Two associated TB/COVID-19 co-infection deaths were reported in men who required invasive mechanical ventilation; one of them was a man living with HIV. Anti-TB treatment was started in all cases. Systemic steroids were indicated for patients with moderate to severe COVID-19 pneumonia according to the doctor’s assessment and criteria.

### 3.3 The COVID-19 pandemic in 2020 decreased the test diagnostics of tuberculosis

**Figure 3:**
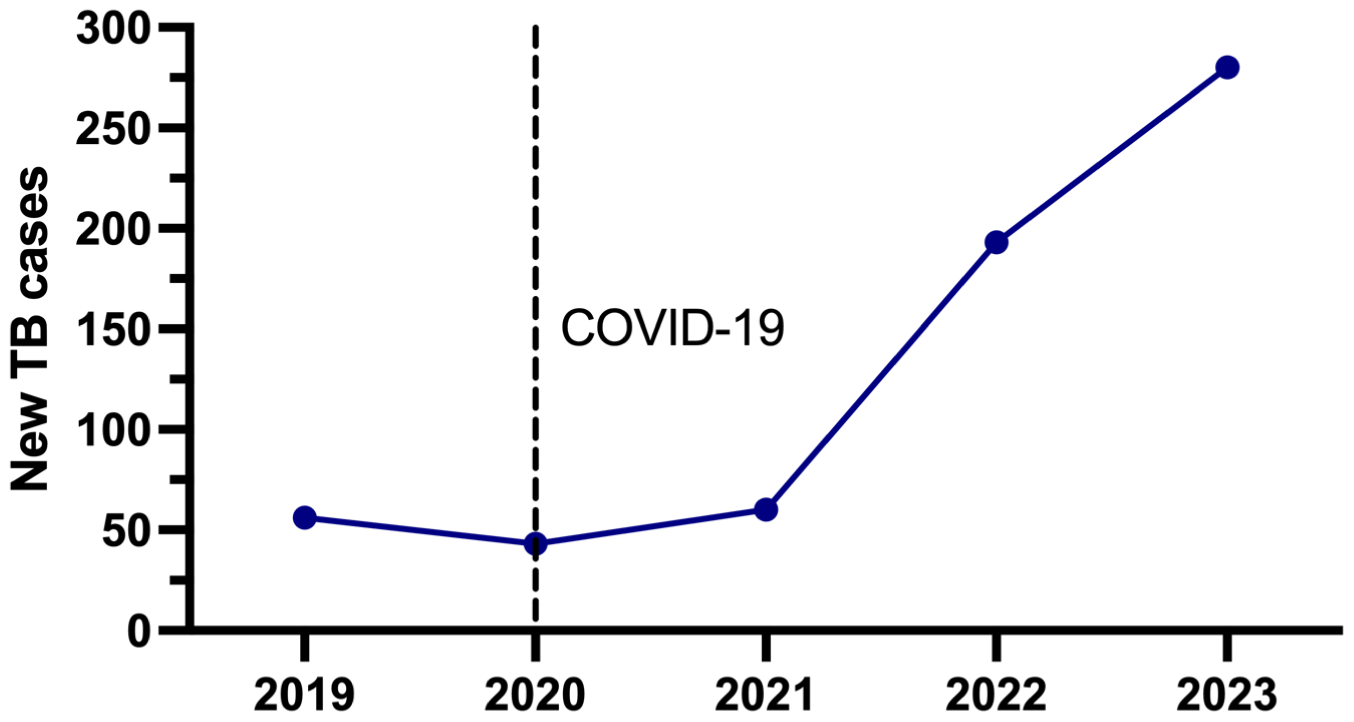
Number of all TB new cases reported in the surveillance national platform from 2019 to 2023. The black dotted line delimits the start of the COVID-19 pandemic in 2020 year.

In accordance with national regulations, new TB cases treated at INER must be reported on the national surveillance platform, encompassing both hospitalized and outpatient individuals. Figure 3 illustrates that reporting new TB cases at our institute significantly increased in 2022 and even more in 2023. For instance, in 2020, the report indicated 43 cases, while in 2023, it showed 279, reflecting an increase of over 600%. Additionally, 2019 is included as a single pre-pandemic reference.

A summarized description of the demographic and clinical information focused on patients newly diagnosed with TB is in Table 4. Data of patients (2019–2023) showed that the average age was 44.4 years, including 395 males (63%), and the most frequent comorbidity in our population was diabetes with 32.4% (205), followed by HIV co-infection 12.1% (77), malnutrition 8.5% (54), alcoholism 7.2% (31) and smoking 6.9% (32). It should be noted that during the COVID-19 pandemic in 2020, the care of patients with TB coinfected with HIV infection was higher (25.5%) compared to the pre-and post-pandemic years.

**Table 4:** Demographic and clinical characteristics of patients corresponding to new TB cases reported yearly.

Year	2019	2020	2021	2022	2023	All TB new cases
TB new cases	67	43	58	191	279	632
Age, mean (SD)	40.0 (±18.5)	44.0 (*±*20.6)	45.4 (± 17.25)	43.8 (± 18.8)	45.70 (± 18.6)	44.4 (± 18.7)
Male, *n* (%)	39 (64)	24 (56)	32 (55)	121 (63)	179 (64)	395 (63)
Diabetes, *n* (%)	24 (39.3)	12 (27.9)	24 (41.3)	56 (29.3)	89 (31.8)	205 (32.4)
HIV, *n* (%)	7 (11.4)	11 (25.5)	6 (10.3)	17 (8.9)	36 (12.9)	77 (12.1)
Malnutrition, *n* (%)	4 (6.5)	4 (9.3)	8 (13.7)	17 (8.9)	21 (7.5)	54 (8.5)
Smoking, *n* (%)	3 (4.9)	2 (4.6)	7 (12.0)	12 (6.28)	20 (7.1)	44 (6.9)
Alcoholism, n (%)	4 (6.5)	5 (11.6)	6 (10.3)	8 (4.1)	23 (8.2)	46 (7.2)

Data are shown as number of patients (%) or mean (± standard deviation, SD). Data show the analysis from the 2019–2023 TB cases electronically recorded and provided by the Epidemiological Surveillance Department.

**Figure 4:**
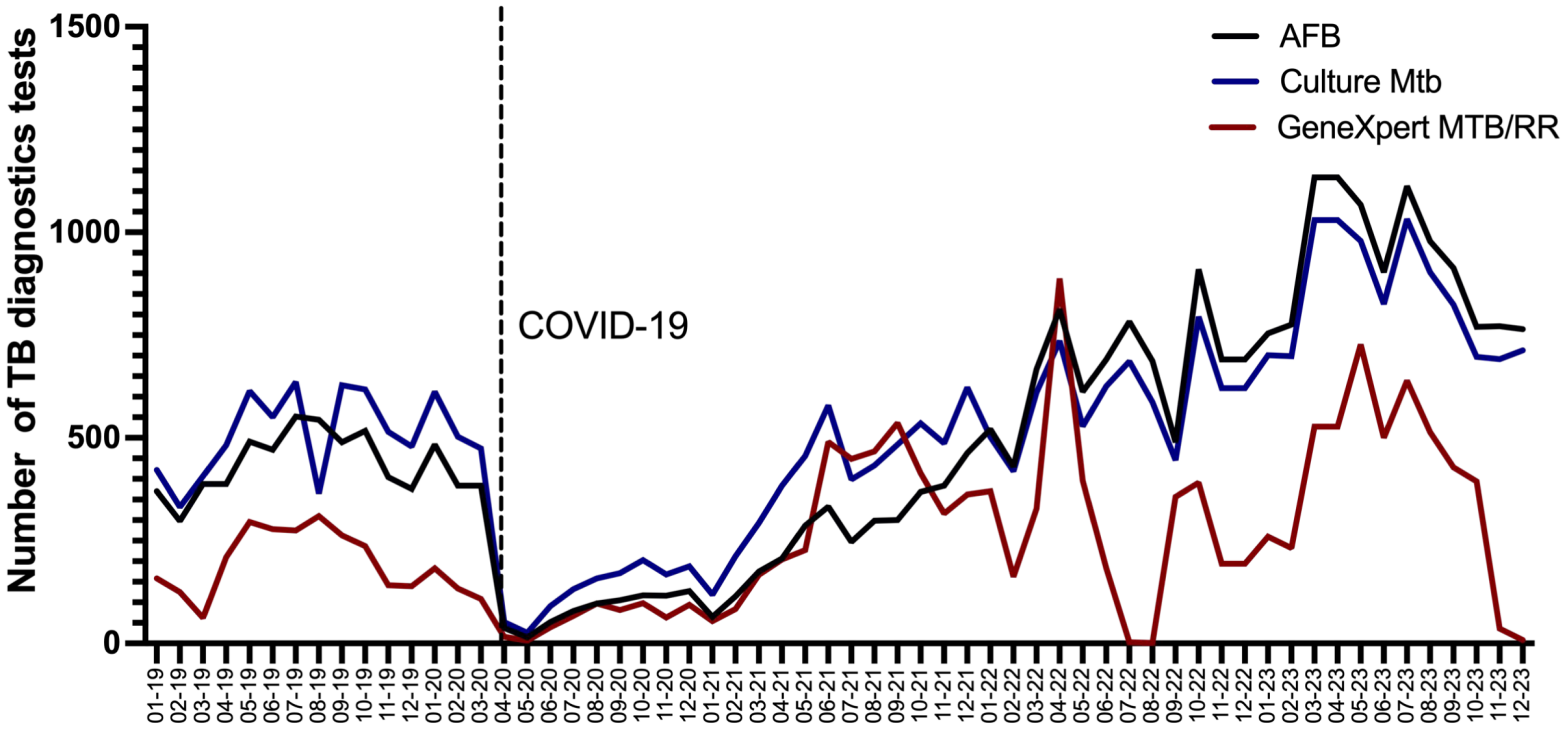
Number of TB diagnosis tests realized during pre-pandemic and post-pandemic COVID-19 2019–2023 per month in a tertiary care of health in Mexico. The black dotted line delimits the start of the COVID-19 pandemic in 2020 year.

The records of the diagnostic tests for TB detection carried out in the INER Microbiology Laboratory from 2019 to 2023 were collected and analyzed. The diagnostic tests included were the number of mycobacterial cultures, GeneXpert MTB/RR Ultra, and AFB (Figure 4; Table 5). Data show that diagnostic tests decreased abruptly in April 2020 after the health emergency decree in Mexico. However, in 2020, our center, a reference laboratory in Mexico City, continued to perform diagnostic tests for TB in a smaller number of patients whom the outpatient clinic area and other hospitals monitored. Noteworthy, there was a significant increase from the first months of 2022, when the hospital conversion in the INER began, which is greater than the pre-pandemic levels.

**Table 5:** Total number of tuberculosis diagnostics tests per year from 2019 to 2023.

Test	2019	2020	2021	2022	2023
Culture of *Mycobacterium tuberculosis*	6,048	2,778	5,004	7,178	10,126
PCR GeneXpert MTB/RR	2,495	987	3,770	3,467	4,792
AFB Smear microscopy	5,288	1998	3,244	7,985	11,078

The diagnosis was performed through a Ziehl-Neelsen staining (AFB, acid-fast-bacilli), Mycobacteria culture (culture Mtb), and GeneXpert® MTB/RR Ultra to identify Mtb and resistance to rifampicin by RT-qPCR.

In 2023, the number of diagnostic tests for TB nearly doubled compared to 2019. This growth is estimated to continue in the coming years, driven by increased outpatient care for respiratory diseases at INER and intensified case-finding efforts. As shown in Figure 4, the GeneXpert MTB/RR test was widely used for TB diagnosis; however, it was unavailable at our institute during mid-2022 and at the end of 2023. Despite this limitation, TB diagnostics keep with AFB studies and mycobacterial culture, which experienced a significant increase in usage.

## 4 Discussion

Despite policies implemented by the WHO and countries’ efforts to control and eradicate TB, the COVID-19 pandemic changed all traced plans. Emergency overwhelmed and overtaken our health care services, directly affecting attending TB patients. WHO emphasized that substantial disruptions to TB case detection for 2020 and 2021, a period critical to the COVID-19 pandemic, reflect an imbalance in the offer and demand for TB diagnostic and treatment services. That could be due to the reduction of the health system’s capacity to continue providing services, reduced ability to seek care by restrictions on movement, concerns about the risks of going to healthcare facilities during a pandemic, and the similarities of symptoms related to TB and COVID-19 (5).

Unfortunately, the TB mortality rate during the COVID-19 pandemic is inaccurate due to the absence of reliable measurement of TB cases and TB mortality by epidemiological surveillance systems in low-and middle-income countries. WHO estimated a 10% window of under-diagnosis of TB; however, it could be higher (5).

Our study found that the COVID-19 pandemic negatively impacted TB patient attendance, particularly among outpatients. Although there was a reduction in overall cases during the pandemic, the proportion of severe TB cases increased. This behavior likely reflects the lack of timely and thorough follow-up for TB patients during the pandemic. Approximately 3.4% of TB patients require critical care, with a mortality rate of up to 48% in this group (33). Additionally, meningeal TB cases are particularly severe, with mortality rates exceeding 60% (30).

In the first pandemic year, we observed a reduction of 76% in the number of attending TB cases (2020 compared to 2019), which is in concordance with other reports. For instance, in India, a country with the highest incidence of TB, notifications for new TB cases drastically declined by 69% during the lockdown months of the pandemic, and the loss of follow-up patients increased by 65% (34). In tertiary-level hospitals in Greece, the number of daily admissions by TB was reduced by 25% in 2020 compared to the two previous years (35). Studies of other countries show similar behavior in diminishing cumulative outpatient visits or TB clinic visits (36–40).

Our analysis reaffirms the relevance and compromise of INER as a reference center for TB diagnosis. Although the number of requested diagnosis tests decreased in 2020 (58.3%), it increased in 2022 and 2023 (34.7 and 88%, respectively) compared to 2019. This data concords with the increase in outpatient care cases and the increase in the processing of samples from other hospitals. Therefore, efforts should also be focused on addressing the demand for diagnosis with molecular tests and detecting cases resistant to anti-TB drugs.

According to several reports, the COVID-19 pandemic substantially impacted the diagnosis and treatment of TB; globally, there is evidence of TB treatment failure (34, 38, 41). Nevertheless, some countries could maintain their TB response (42, 43). The TB management could have been affected by the provisional reassignment of TB services to other medical services (25–27). Furthermore, we believe that some new TB cases diagnosed in outpatient care and emergency rooms post-pandemic were a result of patients initially seeking medical attention for suspected COVID-19. After a thorough evaluation, these patients were subsequently diagnosed with TB.

Concerns about the risk of synergy between TB and COVID-19 during the health crisis have been discussed in several reports. TB/COVID-19 coinfection has been associated with worse clinical outcomes and with up to a 2-fold increase in mortality. However, systematic verifications have found high heterogeneity in the studies of the disease (44, 45).

Given this landscape, international efforts were made to determine the global prevalence and mortality risk due to co-infection. A meta-analysis reported a mortality rate between 13.9 and 17.5% (32). In a global cohort study of TB/COVID-19 co-infection, a mortality rate of 10.8% was reported, with risk factors including advanced age, male sex, comorbidities such as HIV, chronic obstructive pulmonary disease, diabetes, renal failure, supplemental oxygen, and invasive mechanical ventilation. This study also reported that 71% of patients with COVID-19 evolved satisfactorily (46).

Compared with previous reports from countries with high TB prevalence, such as South Africa, India, and Brazil (31), our study identified a low prevalence of TB/COVID-19 co-infection, eight cases, and two associated deaths. This low prevalence of co-infected patients may not reflect accurate statistics because our hospital was an exclusive referral center for COVID-19 cases, specifically for moderate and severe cases. Moreover, TB patients were referred to general hospitals for care, and for hospitalized COVID-19 patients, TB screening tests were only ordered based on the physician’s clinical assessment.

Although the evidence is limited, cases have also been reported in which COVID-19 infection has led to TBD (active disease) in cases with previous TBI, mainly in severe cases of COVID-19 (47, 48). In our center, tests to detect TBI have not been performed before screening patients admitted with COVID-19, so the proportion of cases of TBI/COVID-19 is unknown. However, new studies are being carried out to answer these questions that remain of interest, mainly in high-risk groups for disease activation, such as patients living with HIV, or cancer, and those using anti-TNF biological treatment.

Despite all the problems caused by the COVID-19 pandemic, we can use this knowledge to positively impact TB patient care. Telemedicine and telehealth development were improved to benefit patients and mitigate the effects associated with the disruption of healthcare systems. The use of telehealth increased in various populations, and evidence was created about the usefulness of the use of these technological tools for monitoring the treatment of TB patients, demonstrating that video-DOT (directly observed therapy) was effective and successful as previously reported by studies where it was implemented video-DOT (49, 50). In addition, other studies evaluated the cost-effectiveness of performing TB screening in patients with respiratory symptoms after a negative COVID-19 test to maintain TB detection during the pandemic in high-TB-load countries (51).

Due to the use of the same platform shared for the diagnosis of TB and COVID-19 (real-time PCR), new algorithms and diagnostic tools were developed for the integrated test of TB and COVID-19 in countries with a high burden of TB (52). Our center has research focused on TB as part of the institutional vision and mission; thus, several protocols to find responses to immune response, diagnosis, and treatment of TB patients are systematically performed; moreover, aboard an integrative training of specialists in respiratory diseases, medical and research areas designed a teaching-learning program to strengthen the formation of medical residents and contribute to achieving the main goal proposed by WHO in TB eradication.

Considering the data analyzed from the pre-and post-pandemic periods, along with the increasing demand for TB care in our center, we could contemplate future actions regarding TB clinical care, strategies for researching the immunopathogenesis of TB, and how to implement the care program for TB patients effectively.

We propose several key actions to prevent diagnostic delays and enhance the care of severe TB cases, particularly for patients treated in emergency settings, to reduce mortality. These include:

- Expanding screening efforts to identify latent TB infection (LTBI) and active TB disease among household contacts and relatives of recently diagnosed patients, helping prevent the rise in new cases.
- Implementing telehealth services to facilitate video consultations, increasing access to follow-up care and improving patient monitoring.
- Strengthening the TB healthcare workforce by increasing the number of trained personnel dedicated to diagnosing new cases, providing follow-up care, and managing complex TB cases.
- Enhancing continuous training programs for healthcare professionals to improve diagnostic sample processing and ensure rigorous epidemiological surveillance for better TB case management.
- Optimizing rehabilitation programs to improve the care and quality of life for patients with post-tuberculosis lung disease (PTLD).
- Advancing research on the immunopathogenesis of severe TB could lead to better-targeted treatments.
- Exploring novel diagnostic biomarkers to improve early detection, particularly for high-risk patients.

These actions will strengthen TB control efforts, improve patient outcomes, and enhance preparedness for future public health challenges.

Furthermore, the recently released Global Plan 2023–2030 aims to eliminate post-pandemic TB as a public health problem by 2030. Its key goals include expanding TB diagnosis and service delivery through a patient-centered approach, with active collaboration from communities and the private sector (53).

Additionally, the successful strategies used in the fight against COVID-19 should serve as a model for TB control. The pandemic demonstrated how health personnel, administrators, and leaders can mobilize resources, leverage technology, and dedicate time and effort to addressing urgent public health crises. Applying similar approaches to TB could significantly improve diagnosis, treatment, and overall public health impact.

This study has some limitations that should be considered for a balanced interpretation of the findings. This descriptive analysis was conducted using data from electronic records, and the complete record of newly diagnosed TB cases is limited to 2019, just 1 year before the COVID-19 pandemic. Additionally, the study analyzed data from only a tertiary care health center, where the patients typically exhibit more complicated clinical manifestations or chronic tuberculosis, which may explain the higher ratio of deaths among TB patients. Despite being a retrospective study with a limited number of cases, it is important to note that the analysis provides insights into the pandemic’s behavior and its impact on newly diagnosed TB cases. Our records indicate that the rise in post-pandemic TB patients attending our hospital aligns with trends observed in other health centers and global reports.

## 5 Conclusion

This study found that TB diagnosis and control were significantly impacted during the COVID-19 pandemic, similar to trends observed in other countries. However, post-pandemic efforts at INER have led to the implementation of telehealth services, reinforcement of human resources dedicated to respiratory diseases, and improvements in diagnostic testing.

Based on these findings, we propose key recommendations and future directions for the post-pandemic era. Strengthening human resources is essential, ensuring adequate healthcare personnel to support medical care, epidemiological surveillance, and microbiology laboratories for timely TB diagnosis. In this regard, hospitals and national TB programs must anticipate and prepare for an increased demand for TB diagnostic services, ensuring sustained investment in workforce capacity and diagnostic infrastructure to prevent future disruptions in TB control.

## Data Availability

The original contributions presented in the study are included in the article/supplementary material, further inquiries can be directed to the corresponding authors.
